# Disseminated *Mycobacterium genavense* Infection in Patient with Adult-Onset Immunodeficiency

**DOI:** 10.3201/eid2307.161677

**Published:** 2017-07

**Authors:** Takanori Asakura, Ho Namkoong, Takuro Sakagami, Naoki Hasegawa, Kiyofumi Ohkusu, Akira Nakamura

**Affiliations:** Japan Society for the Promotion of Science, Tokyo, Japan (T. Asakura);; Keio University School of Medicine, Tokyo (T. Asakura, H. Namkoong, N. Hasegawa);; Eiju General Hospital, Tokyo (H. Namkoong);; Niigata University Graduate School of Medical and Dental Sciences, Niigata, Japan (T. Sakagami);; Tokyo Medical University, Tokyo (K. Ohkusu);; Asahi General Hospital, Chiba, Japan (A. Nakamura)

**Keywords:** Mycobacterium genavense, adult-onset immunodeficiency, bacteria, Tuberculosis and other mycobacteria, Japan, nontuberculous mycobacteria, HIV

## Abstract

We report a case of disseminated *Mycobacterium genavense* infection resulting from neutralizing anti–interferon-γ autoantibodies in the patient. We identified *M. genavense* targeting the *hsp65* gene in an aspiration specimen of the lymph node. Adult-onset immunodeficiency caused by neutralizing anti–interferon-γ autoantibodies, in addition to HIV infection, can lead to disseminated nontuberculous mycobacterial infection.

*Mycobacterium genavense* is a ubiquitous nontuberculous mycobacteria (NTM), first described as a human infection in the 1990s as a primary cause of fatal disseminated infection in HIV-infected patients with low CD4 counts (*1*). *M. genavense* also is recognized as an opportunistic pathogen in patients without HIV who have severe immunodeficiency, such as that after solid-organ or hematopoietic stem cell transplantation or immunosuppressive therapy (*2*,*3*). The diagnosis of *M. genavense* infection is clinically challenging because of the difficulties in routinely culturing the organism and the absence of specific symptoms, even in fatal infections. Therefore, diagnosing *M. genavense* infection in patients without known evidence of immunodeficiency is particularly difficult. We report a previously healthy 66-year-old man with multiple lymphadenopathies in whom disseminated *M. genavense* infection resulting from the presence of neutralizing anti–interferon-γ (anti–IFN-γ) autoantibodies was diagnosed.

In November 2015, the patient sought care at Asahi General Hospital (Chiba, Japan) for a 2-week history of right-side neck swelling and abdominal pain. His vital signs were within reference ranges. Except for right cervical lymphadenopathy, findings on physical examination were unremarkable. HIV antibodies were undetectable, and CD4/CD8 lymphocyte counts were within reference ranges. No mediastinal or lung involvement was detected on chest computed tomography (CT) scan. Gallium-67 single-photon emission CT/CT revealed high-intensity accumulation of the right cervical and ileocolic lymph nodes (Technical Appendix Figure). Acid-fast bacilli (AFB) staining of the lymph-node aspiration specimen yielded positive results; however, findings on solid media culture and PCR for detecting *M. tuberculosis*, *M. avium*, and *M. intracellulare* were negative. After a 6-week outpatient follow-up, the patient returned with newly developed right axillary lymphadenopathy. An aspiration specimen of the lymph node showed positive AFB staining and was submitted for molecular biologic analysis. *M. genavense* was identified on amplification and sequencing analysis targeting the *hsp65* gene (*4*). We strongly suspected neutralizing anti–IFN-γ autoantibodies as the cause because the whole blood IFN-γ level with mitogen stimulation was low, as determined using an IFN-γ–releasing assay (QuantiFERON TB 3G; Cellestis, Carnagie, VIC, Australia). A high serum-neutralizing anti–IFN-γ autoantibody titer and inhibited STAT1 (signal transducer and activator of transcription 1) phosphorylation through IFN-γ stimulation in the leukocytes were confirmed, leading to a diagnosis of disseminated *M. genavense* infection. Clarithromycin, ethambutol, rifampin, and amikacin were administered. Lymphadenopathy improved after 6 weeks, and amikacin was discontinued. No relapse occurred during 16 months of treatment.

Recent studies have described disseminated NTM infection in patients in Asia with adult-onset immunodeficiency resulting from neutralizing anti–IFN-γ autoantibodies (*5*–*7*). Disseminated infection mainly involves the lymph nodes, followed by the osteoarticular system, bone, lungs, and skin (*6*,*7*). The pathogen comprises rapidly and slowly growing mycobacteria; *M. avium* complex and *M. abscessus* are the most frequently detected. Although the long-term outcome is unclear, most patients need long-term antimicrobial therapy, and some relapses occur after treatment discontinuation (*6*,*7*). Adjuvant rituximab therapy has been used for refractory disease (*8*).

Although disseminated *M. genavense* infections formerly only were known to occur in HIV-infected patients, the epidemiologic shift to infections in patients without HIV reflects the introduction of combination antiretroviral therapy and increasing use of immunosuppressive agents (*2*). In 2 previous series comprising 14 HIV-negative patients with *M. genavense* infection, most patients had known evidence of immunodeficiency; of the 12 patients treated with immunosuppressive agents, 5 had sarcoidosis, 5 were solid-organ transplantation recipients, 1 had non-Hodgkin lymphoma, and 1 had rheumatoid arthritis. Only 2 patients were identified with adult-onset innate immunodeficiency (*2*,*3*); 1 patient had innate interleukin-12 receptor deficiency and 1 had idiopathic CD4 lymphocytopenia.

Needle aspirates and tissue biopsy provide higher NTM diagnostic yields than does swab sampling but are insufficiently sensitive. Therefore, less frequently encountered mycobacterial species are identified by gene sequencing, reverse hybridization, and high-performance liquid chromatography (*9*). Moreover, the identification of *M. genavense* infection using standard mycobacterial culture methods is difficult. Acidified solid media testing with blood and charcoal is probably the most suitable method (*10*); however, accurate diagnosis requires additional molecular biologic analysis, such as amplification and sequencing of the 16S ribosomal RNA, *hsp65*, or *rpoB* genes. In this case, we identified *M. genavense* using a direct molecular biologic method for aspiration specimens from the lymph node.

Little is known about death among HIV-negative patients with *M. genavense* infection, although some patients reportedly have died (*2*,*3*). Although their conditions eventually improve, despite a lack of early identification of *M. genavense*, delayed diagnosis might influence death. Direct molecular biologic methods could better identify *M. genavense* infection and improve prognosis.

We report a case of disseminated *M. genavense* infection resulting from neutralizing anti–IFN-γ autoantibodies in the patient. *M. genavense* infection should be considered in the differential diagnosis of mycobacteria detected with AFB staining but not with culture, even in patients without known evidence of immunodeficiency. Adult-onset immunodeficiency acquired by neutralizing anti–IFN-γ autoantibodies, in addition to HIV infection, can lead to disseminated NTM infection.

Technical Appendix Gallium-67 single-photon emission computed tomography/computed tomography showing high-intensity accumulation of *Mycobacterium genavense* in the right cervical (A) and ileocolic (B) lymph nodes of a 66-year-old previously healthy man.
