# Supplementary material for: Disseminated Mycobacterium genavense Infection in Patient with Adult-Onset Immunodeficiency
Source: Emerg Infect Dis. 2017 Jul;23(7):1208–10. doi: 10.3201/eid2307.161677 (PMC5512497; doi:10.3201/eid2307.161677)
Supplement: Technical Appendix — Gallium-67 single-photon emission computed tomography/computed tomography showing high-intensity accumulation of Mycobacterium genavense in the right cervical (A) and ileocolic (B) lymph nodes of a 66-year-old previously healthy man. [file 16-1677-Techapp-s1.pdf]

Article DOI: https://dx.doi.org/10.3201/eid2307.161677

Disseminated *Mycobacterium genavense* Infection in Patient with Adult-Onset Immunodeficiency

Technical Appendix.

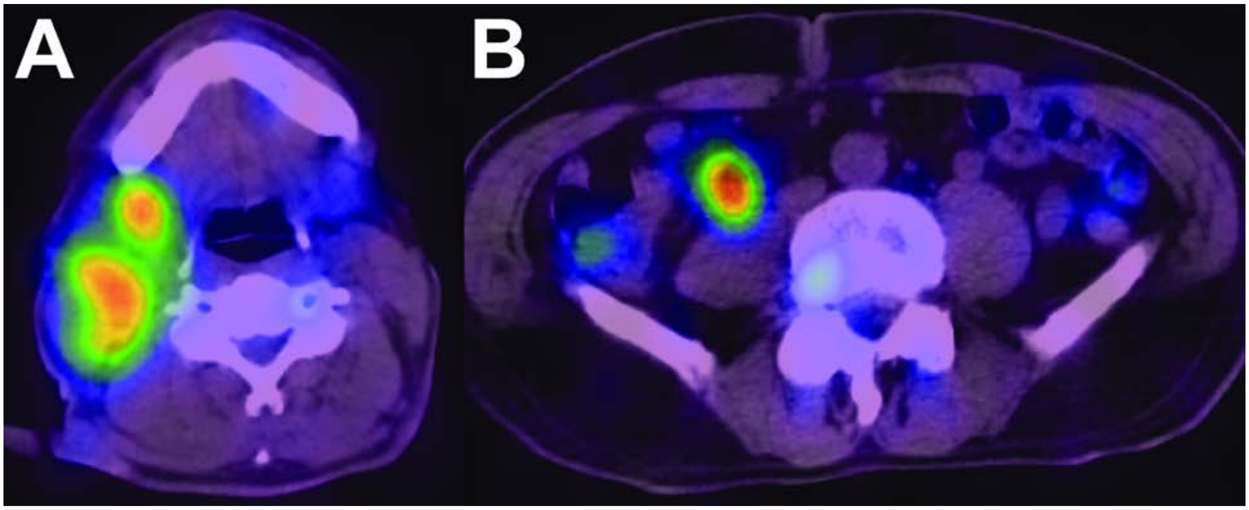

Technical Appendix Figure. Gallium-67 single-photon emission computed tomography/computed tomography showing high-intensity accumulation of *Mycobacterium genavense* in the right cervical (A) and ileocolic (B) lymph nodes of a 66-year-old previously healthy man.
